# Roles of Rac1 and Rac3 GTPases during the development of cortical and hippocampal GABAergic interneurons

**DOI:** 10.3389/fncel.2014.00307

**Published:** 2014-09-25

**Authors:** Ivan de Curtis

**Affiliations:** Cell Adhesion Unit, Division of Neuroscience, San Raffaele Scientific Institute and Vita-Salute San Raffaele UniversityMilano, Italy

**Keywords:** cortex, effector, GEF, hippocampus, interneuron, Rac GTPase

## Abstract

Rac GTPases are regulators of the cytoskeleton that play an important role in several aspects of neuronal and brain development. Two distinct Rac GTPases are expressed in the developing nervous system, the widely expressed Rac1 and the neural-specific Rac3 proteins. Recent experimental evidence supports a central role of these two Rac proteins in the development of inhibitory GABAergic interneurons, important modulatory elements of the brain circuitry. The combined inactivation of the genes for the two Rac proteins has profound effects on distinct aspects of interneuron development, and has highlighted a synergistic contribution of the two proteins to the postmitotic maturation of specific populations of cortical and hippocampal interneurons. Rac function is modulated by different types of regulators, and can influence the activity of specific effectors. Some of these proteins have been associated to the development and maturation of interneurons. Cortical interneuron dysfunction is implicated in several neurological and psychiatric diseases characterized by cognitive impairment. Therefore the description of the cellular processes regulated by the Rac GTPases, and the identification of the molecular networks underlying these processes during interneuron development is relevant to the understanding of the role of GABAergic interneurons in cognitive functions.

## Introduction

**Figure 1:**
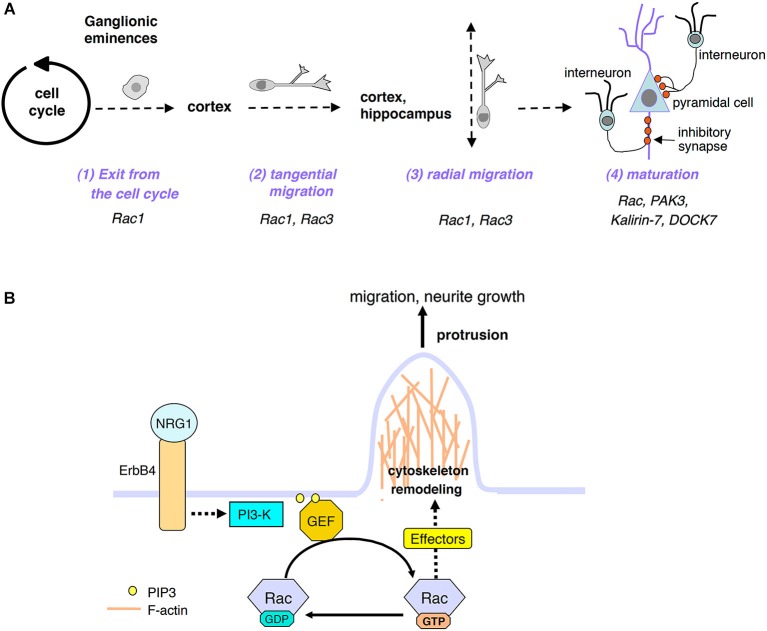
**(A)** Scheme of distinct developmental phases of postmitotic interneuron maturation: after exiting the cell cycle, the GABAergic precursors leave the GE by migrating tangentially; once they have reached their final destination (cortex and hippocampus), they will switch to radial migration to position properly. GABAergic cells will then continue to mature by developing axon and dendrites, and by forming inhibitory synapses with pyramidal cells and with other interneurons (not shown). Some of the proteins relevant to these processes and described in this review are indicated. **(B)** Model for the activation of Rac GTPases by extracellular signals important for interneuron migration and maturation. See text for details.

Inhibitory γ-aminobutyric acid (GABA)ergic interneurons modulate brain functions (Batista-Brito and Fishell, 2009). Several studies indicate that abnormal development of these neurons causes the unbalance of excitatory/inhibitory signals, which may cause the neural and intellectual impairment observed in disorders such as epilepsy, autism and schizophrenia (Lewis et al., 2005; Orekhova et al., 2007; Lawrence et al., 2010; Sebe and Baraban, 2011; Velíšek et al., 2011; Le Magueresse and Monyer, 2013). Most cortical and hippocampal GABAergic interneurons have a common origin: they are born in the ganglionic eminences (GE), transitory embryonic structures in the developing ventral telencephalon (Danglot et al., 2006; Wonders and Anderson, 2006; Tricoire et al., 2011). GABAergic precursors leave the GE migrating tangentially along three tangential streams in the marginal zone (MZ), subplate (SP), or subventricular zone (SVZ), towards their cortical/hippocampal destination, where they switch to radial migration to populate the different layers of these areas (Tanaka et al., 2006; Martini et al., 2009; Figure 1A). Many studies have addressed the mechanisms regulating the development of GABAergic interneurons, and led to the identification of transcription factors and extracellular cues driving their differentiation and migration (Hernández-Miranda et al., 2010). Conversely, the knowledge of the intracellular mechanisms driving the different phases of interneuron development is limited.

Rho family GTPases are important regulators of cytoskeletal dynamics. Among them, Rac proteins are critical in several aspects of neuronal development (de Curtis, 2008; Heasman and Ridley, 2008). This review focuses on studies supporting the role of Rac proteins, their regulators and effectors on the development of cortical and hippocampal GABAergic interneurons. These cells form inhibitory synapses that modulate the output from excitatory neurons and other interneurons. These interneurons go through a sequence of developmental events including exit from the GEs, tangential and radial migration, and late maturation steps including extension and branching of axons and dendrites, target recognition, and establishment of inhibitory synapses. Each developmental step requires the association of extracellular signals with specific surface receptors, resulting in the activation of proper intracellular signals. Here, I will present the available evidence on the role of Rac proteins and of proteins functionally-associated to these GTPases in different steps of the development of cortical/hippocampal GABAergic cells.

## Rac GTPases regulate the development of interneurons

Rho family GTPases are molecular switches that regulate several processes including the organization of the actin cytoskeleton (Hall, 1998). The Rho family is subdivided into groups of highly homologous proteins. One of these groups includes the Rac GTPases. There are three genes in vertebrates encoding the Rac1, Rac2 and Rac3 proteins that share high sequence identity (88–92%), and a fourth gene encoding the Rac-related RhoG protein (about 70% identity with Rac1). While Rac2 is specifically expressed in hematopoietic cells (Didsbury et al., 1989), Rac1, Rac3 (initially identified as Rac1B in chicken), and RhoG are expressed in neurons (Moll et al., 1991; Vincent et al., 1992; Corbetta et al., 2005). Rac3 expression is often, but not always overlapping with that of Rac1 (Corbetta et al., 2005).

Most studies on neurons have considered Rac1. On the other hand, over the past years we have proposed that both Rac1 and Rac3 need to be considered to fully understand the role of Rac in neuronal development and function (de Curtis, 2008). While Rac1 is widely expressed from early embryonic development, Rac3 is mainly, if not exclusively expressed in developing neurons. Our findings have shown that the two GTPases cooperate to build up a functional brain (Corbetta et al., 2009). In this direction, a number of recent studies have emphasized the importance of analyzing both Rac1 and Rac3 during the development of GABAergic interneurons.

## Rac GTPases in early development and interneuron migration

The first two studies on the role of Rac proteins in the development of cortical interneurons suggested that Rac is not directly implicated in the development of postmitotic migratory interneurons. The earlier conditional knockout of Rac1 in the pre-migratory neural progenitors of the ventricular zone (VZ) of the telencephalon causes several defects, including a failure of the tangential migration of interneurons from the GEs (Chen et al., 2007; Figure 1A). In apparent contrast, the same study shows that later deletion of Rac1 in postmitotic migratory cells exiting the VZ and into the SVZ of the ventral telencephalon does not affect tangential migration. Previous findings have shown that neurons have to reach a proper differentiation state to use the machinery needed for migration (Kuhar et al., 1993; Hatten, 1999). Based on this assumption, the authors explain their apparently contrasting findings by proposing that the defect in migration observed after early Rac1 deletion is due to Rac1-dependent GABAergic progenitor-specific functions required to establish the competency of migration at later stages, and not to a primary abnormality of cell motility, as indicated by their other finding that Rac1 is dispensable for interneuron motility during the migratory phase. Similar results were obtained by a more recent study addressing the role of Rac1 on interneuron progenitors from the medial ganglionic eminence (MGE; Vidaki et al., 2012). In this study, Rac1 ablation in postmitotic GABAergic precursors does not affect the number of MGE-derived cortical interneurons, while earlier deletion in proliferating MGE progenitors causes a reduction in the number of MGE-derived cortical interneurons. This loss is due to a defect in the exit of the precursors from the cell cycle (Figure 1A). This study has highlighted a cell-autonomous, stage-specific requirement of Rac1 activity in the proliferating precursors. Altogether, these studies show that the conditional deletion of Rac1 impairs the early phases of interneuron differentiation, while the migration of cortical interneurons is not affected if Rac1 is deleted after the progenitors have left the MGE to migrate into the pallium. To explain these results, it has to be considered that mammals and other vertebrates express the neural-specific Rac3/Rac1B GTPase (Albertinazzi et al., 1998, 2003) during the late development of peripheral and central nervous system neurons (Corbetta et al., 2005). Rac3 protein expression is developmentally regulated, with a peak at time of neurite branching and synaptogenesis (Bolis et al., 2003). Therefore, Rac1 deletion in MGE dividing progenitors, at a time when Rac3 is not or very poorly expressed, is sufficient to indirectly affect migration. On the other hand, the lack of effects on migration after Rac1 is deleted in postmitotic migratory precursors may be explained by compensation with Rac3, which is expressed at these later stages. Since RhoG is also expressed in the nervous system, RhoG may also compensate for the lack of Rac1 GTPase at later stages. So far though no studies are available supporting a role of RhoG in the development of interneurons.

The hypothesis that Rac3 may compensate for the lack of Rac1 in supporting the tangential migration of GABAergic precursors is endorsed by recent studies analyzing the effects of the double knockout of the two GTPases on interneuron development. The generation of double mutants obtained by combining the full deletion of Rac3 (Rac3KO) with the conditional deletion of Rac1 in postmitotic neurons by the Synapsin-I-Cre results in mice that are neurologically impaired, with spontaneous epileptic seizures (Corbetta et al., 2009). Interestingly the transcript for Rac3 is detected in PV-positive interneurons, and the double knockout mice show a strong loss of MGE-derived PV-positive cells in the cortex and hippocampus (Vaghi et al., 2014). While apoptosis does not appear to contribute to this loss, the defects observed in the MGE-derived precursors undergoing tangential and radial migration indicate that Rac GTPases are important for the migration of postmitotic interneurons (Figure 1A), and that the migratory defects may underlie the loss of cortical and hippocampal interneurons in the postnatal brain of the double knockout mice. Loss of postnatal cortical interneurons and migratory defects of the precursors have been confirmed in a later study analyzing the contribution of Rac1 and Rac3 in the development of GABAergic cells (Tivodar et al., 2014). In this study, when interneurons depleted of both Rac proteins were placed in culture, they revealed gross defects in the cytoskeletal organization; they showed severe morphological alterations including an increased number of neurites per neuron, shorter axons, and abnormal growth cones when compared to either wildtype of Rac1KO cells. On the other hand, our analysis *in vivo* indicated a defect in the later maturation of double mutant interneurons (Vaghi et al., 2014). This defect correlates with evident alteration of the electrophysiological properties of cortical and hippocampal circuits in double knockout mice, as shown by the increased excitability and decreased spontaneous inhibitory currents in the cortical and hippocampal pyramidal cells of these animals.

Of note, the milder loss of PV-positive cells in the cortex and hippocampus of single Rac1 or Rac3 knockout mice (Vaghi et al., 2014), on the one hand demonstrates the functional redundancy of the two Rac proteins, on the other hand shows that during the development of cortical interneurons each Rac has specific roles that can not be compensated by the other GTPase.

## The control of cortical interneuron maturation by Rac-dependent mechanisms

While the direct implication of Rac GTPases in the early development of cortical and hippocampal interneuron precursors is well established, the involvement of these proteins in later steps of interneuron maturation, after the cells have reached their final destination, is still limited. Analysis of double knockout mice suggests that Rac depletion also influences the later maturation of interneurons, as detected by the reduction of the PV-positive signal in the neuropilum of the stratum pyramidale of the hippocampus, and of the inhibitory VGAT/GAD67-positive GABAergic presynaptic terminals around hippocampal and cortical pyramidal cells (Vaghi et al., 2014). The decrease in presynaptic input corresponds to electrophysiological alterations of cortical and hippocampal circuitry in these mice, which have hyperexcitable pyramidal neurons that also show a significant decrease of spontaneous inhibitory post-synaptic currents. Although these data show that Rac1 and Rac3 are required for the development of hippocampal and cortical inhibitory circuits, the contribution of either Rac remains an interesting topic for further analysis. To date specific molecular defects linked to the loss of either Rac1 or Rac3 GTPases have not been identified. Still, specific behavioral effects have been described in Rac3KO mice, which have a generalized hyperactivity that is not linked to detectable cognitive deficits (Corbetta et al., 2008). This phenotype is specific for Rac3, since it is not prevented by the endogenous Rac1 protein still present in the Rac3KO mice. Whether this phenotype corresponds to a defect of the inhibitory networks in the Rac3KO mice remains to be established. If so, this will open the possibility to explore the molecular machinery linked to Rac function and underlying the hyperactive/impulsive behavior, phenotypes observed in different neurologic and psychiatric disorders such as ADHD, epilepsy, and autistic disorders (Robinson, 2012; Pincham, 2014).

A number of studies have demonstrated the involvement of Rac effectors or regulators in the development of cortical interneurons (Table 1). The p21-activated serine/threonine kinases PAK1 and PAK3 are members of the PAK family activated by Rac and Cdc42. PAK1 and PAK3 are highly expressed in the brain, and their double knockout causes the reduction of dendritic complexity in cultured hippocampal neurons (Huang et al., 2011). Moreover mutations in the gene for PAK3 are associated with nonsyndromic mental retardation (Ramakers, 2002). Downregulation of PAK3 affects the number of mature spines in hippocampal pyramidal cells (Boda et al., 2004; Thévenot et al., 2011; Dubos et al., 2012), and mice with knockout of PAK3 show cognitive impairment (Meng et al., 2005). PAK3 is also implicated in the maturation of cortical GABAergic cells. PAK3 expression is maintained low in cortical migratory precursors by the homeobox transcription factors Dlx1/2, while it is upregulated in post-migratory MGE- and CGE-derived interneurons. The double knockout of Dlx1/2 causes the premature increase of PAK3 expression in the precursors, leading to untimely development of axons and dendrites (Cobos et al., 2007). These findings suggest that repression of PAK3 expression by Dlx1/2 is critical to prevent neuritogenesis and to promote the migration of the interneuron precursors. This hypothesis is supported by the recent analysis of the role of Dlx1 *in vitro*. In hippocampal cultures, GAD67-positive interneurons specifically express Dlx1, which is not expressed in pyramidal neurons (Dai et al., 2014). Knockdown of Dlx1 enhances dendritic growth in GAD67-positive interneurons, supporting a role of Dlx1 in restricting dendritic complexity. Interestingly, overexpression of PAK3 increases dendritic complexity as observed after Dlx1 silencing. Further support that Dlx1 and PAK3 regulate dendritic branching comes from the observation that exogenous expression of Dlx1 in the excitatory pyramidal cells reduces dendritic complexity, and this reduction is partially rescued by co-expression of PAK3. Altogether, the data support a role of PAK3 in the maturation of dendrites and in the postsynaptic differentiation of excitatory and GABAergic neurons.

**Table 1:** **Proteins functionally associated to Rac GTPases and implicated in the development of cortical/hippocampal GABAergic cells**.

Protein	Gene name	Function related to Rac	Link to disease (reference)	Role in GABAergic cell development
DOCK7	DOCK7	Rac GEF	Epileptic encephalopathy (Perrault et al., 2014)	Development of Chandelier cells
JNK3	MAPK10/JNK3	Member of the JNK family kinases activated downstream of Rac	Epileptic encephalopathy (Shoichet et al., 2006); intellectual disability (Kunde et al., 2013)	JNK1/JNK2: guidance during interneuron migration into the cortex
GIT1	GIT1	ArfGAP interacting with PIX family Rac GEFs	ADHD (Won et al., 2011) Huntingtin aggregation (Goehler et al., 2004)	Unknown: KO affects inhibitory input and PV+ cells
Kalirin-7	KLRN	Rac GEF	Schizophrenia (Hill et al., 2006)	Dendritic growth; potentiation of excitatory postsynaptic terminals
PAK3	PAK3	Effector of Rac/Cdc42	X-linked mental retardation (Allen et al., 1998)	Neuritogenesis, dendritic maturation

Increasing evidence supports the role of Rac GEFs (guanine nucleotide exchange factors) in the development of interneurons. The secreted factor neuregulin-1 (NRG1) interacts with its transmembrane tyrosine kinase receptor ErbB4 to promote the growth of dendrites in mature interneurons. Evidence has been produced that Kalirin-7, a major dendritic Rac GEF (guanine nucleotide exchange factor) of the Trio/Kalirin family, mediates the effects of NRG1/ErbB4 activation on the growth of dendrites in interneurons, and that the phosphorylation of the carboxy-terminus of kalirin-7 is critical for these effects (Cahill et al., 2012). Intriguingly, overexpression of kalirin-7 does not only increase the branching of dendrites of hippocampal CA1 interneurons, but induces also the formation of spine-like structures in these usually aspiny cells (Ma et al., 2008), indicating a role of this Rac GEF in potentiating the formation of excitatory postsynaptic terminals in GABAergic neurons. Interestingly, kalirin-7, NRG1, and ErbB4 are highly and specifically expressed in GABAergic interneurons, and have been associated with schizophrenia (Ma et al., 2001, 2005; Hill et al., 2006; Li et al., 2006; Fazzari et al., 2010; Del Pino et al., 2013; Kasnauskiene et al., 2013). Since the dendritic length of interneurons is reduced in schizophrenia (Kalus et al., 2002), understanding how NRG1/ErbB4 signaling regulates the dendrites of these cells may help clarifying the mechanisms underlying the cortical defects in this disorder.

The positive effects of NRG1/ErbB4-induced signaling on the neurite morphology and synaptic maturation of interneurons are mediated by the phosphoinositide 3-kinase (PI3-kinase) pathway that produces phosphatidylinositol 3,4,5-trisphosphate (PIP3) at the plasma membrane. How the activation of PI3-kinase affects the migration of the precursors and their later differentiation into mature inhibitory neurons is unknown. One possibility is that PIP3 engages the GEFs to activate the Rac’s at the plasma membrane (Han et al., 1998; Ma et al., 1998), where they will cause the rearrangement of the actin cytoskeleton that is required both for the migration of the interneurons and for the maturation of their neurites (Figure 1B). According to this hypothesis, Rac may mediate also the cytoskeletal reorganization required for the stimulation of tangential migration towards the cortex and hippocampus of the MGE-derived GABAergic precursors induced by the binding of the neurotrophins BDNF and NT4 to the tyrosine kinase receptor TrkB (Polleux et al., 2002). As for the NRG1/ErbB4-induced signaling described before, the signaling induced by BDNF and NT4 rapidly activates the enzyme PI3-kinase, as indicated by the strong inhibition of the tangential migration of interneurons by the PI3-kinase inhibitor LY294002.

Another Rac GEF, DOCK7 controls the development of chandelier cells, a specific class of GABAergic interneurons characterized by distinctive axonal terminals that target the axon initial segment of pyramidal cells by forming typical cartridge structures. DOCK7 regulates the development of chandelier cells by physically interacting and enhancing the activity of the receptor tyrosine kinase ErbB4 in a GEF-independent manner (Tai et al., 2014). The loss of DOCK7 function in cortical interneurons may underlie the abnormal development of GABAergic networks that could lead to the seizures observed in individuals with epileptic encephalopathies that carry truncating mutations in the gene for DOCK7 (Perrault et al., 2014).

The activity of the c-Jun N-terminal kinases (JNK) can be regulated by Rac GTPases (Coso et al., 1995). Two different mutations have been identified in a member of this family, JNK3, which cause either mild intellectual disability (Kunde et al., 2013) or severe developmental epileptic encephalopathy (Shoichet et al., 2006). Recently, JNK signaling has been shown to be important to guide the migration of interneurons into the mouse cerebral cortex (Myers et al., 2014). Pharmacological inhibition of JNK signaling on brain sections disrupts the migration of interneurons into the embryonic cortex. Analysis by time lapse showed that cells incubated with JNK inhibitors moved along aberrant trajectories. The involvement of JNK1 and JNK2 was confirmed by similar defects observed in sections from single JNK1 or double JNK1/JNK2 knockout mice. Since no effects of the knockouts were observed on interneuron migration *in vitro*, the authors suggest that JNK signaling is regulating guidance rather than motility in these cells.

Another protein indirectly linked to Rac signaling is G protein–coupled receptor kinase–interacting protein-1 (GIT1), which together with GIT2 is part of a family of scaffold proteins with Arf GTPase activating protein (GAP) activity. GIT proteins form stable complexes with the Rac/Cdc42 GEFs of the PIX family, αPIX, and βPIX (Totaro et al., 2007, 2012). GIT/PIX/PAK complexes can be isolated from neural tissues by binding to activated Rac GTPases (Di Cesare et al., 2000; Albertinazzi et al., 2003). The GIT1 complex plays a central role in the formation of dendritic spines at excitatory synapses: proper localization of the GIT1/βPIX complex at postsynaptic sites by the central synaptic localization domain of GIT1 is fundamental to recruit Rac and PAK activity to stabilize the actin network in dendritic spines (Zhang et al., 2003, 2005). Moreover, the knockout of GIT1 negatively affects the length of dendrites and the density of cortical and hippocampal spines, causing defects in fear response and learning (Schmalzigaug et al., 2009a; Menon et al., 2010). Also αPIX, which like PAK3 is mutated in patients with mental retardation (Ramakers, 2002), contributes to synapse formation (Nodé-Langlois et al., 2006; Ramakers et al., 2012). The hypothesis that the GIT1/PIX/PAK complexes may also contribute to the formation of inhibitory synapses is supported by the finding that GIT1 colocalizes with the inhibitory synaptic marker GAD67 (Zhang et al., 2003).

Less is known about the role in neuronal development of the widely expressed GIT2 protein. GIT2 knockout mice have no evident brain defects, but show anxiety-like behavior as a major neurological symptom (Schmalzigaug et al., 2009b). GIT/PIX complexes are regulating neurite branching in cultured hippocampal neurons, with specific effects of GIT2 and αPIX (Totaro et al., 2012).

Interestingly, it has been recently shown that knockout of GIT1 causes a strong and specific reduction in the inhibitory input and in the number of PV-positive interneurons in the hippocampal CA1 region, where the GIT1 protein is normally expressed (Won et al., 2011). The decrease in Rac/PAK/PIX/GIT signaling detected in the GIT1 knockout brain suggests a functional link between GIT1 and Rac GTPases during the development of inhibitory hippocampal networks. This defect correlates with the appearance of neurological traits associated with human ADHD (Attention deficit hyperactivity disorder). Accordingly, the authors have identified an intronic polymorphism in the human gene for GIT1 that is associated with increased ADHD susceptibility, and that may cause reduced GIT1 expression. Interestingly, this study shows that GIT1-knockout mice show ADHD-like phenotypes, with traits including hyperactivity, enhanced electroencephalogram theta rhythms, and impaired learning and memory. Hyperactivity in GIT1 knockout mice is reversed by amphetamine and methylphenidate, psychostimulants commonly used to treat ADHD.

## Conclusions

The role of Rac1 and Rac3 in the development of cortical and hippocampal GABAergic interneurons has been confirmed by recent studies. Still, substantial work remains to characterize the distinct effects of the two Rac proteins in different developmental steps required for the formation of mature interneurons. Moreover, a number of players has been identified that may regulate or mediate the action of the Rac GTPases in these cells. The wide variety of GABAergic cell types required for normal brain function suggests that specific combinations of regulators and effectors for these GTPases are expected to take part in the maturation and function of distinct types of interneurons. Most of the work on developing interneurons has been performed using mice models, but the fewer studies *in vitro* highlight the importance of flanking the studies *in vivo* with reductionistic approaches entailing explants or primary cultures of interneurons from the GEs, to address the machinery required for their development in simplified experimental systems. The identification of the molecular sets involved in the different phases of interneuron development is expected to give an important contribution to the understanding of different neurological and psychiatric disorders where defects in cortical GABAergic cell development appears to importantly contribute to the pathogenesis.

## Conflict of interest statement

The author declares that the research was conducted in the absence of any commercial or financial relationships that could be construed as a potential conflict of interest.
